# A qualitative content analysis of cannabis-related discussions on Reddit during the COVID-19 pandemic

**DOI:** 10.1371/journal.pone.0304336

**Published:** 2024-06-06

**Authors:** Hannah Reygaerts, Sidney Smith, Lynette M. Renner, Yumary Ruiz, Laura M. Schwab-Reese

**Affiliations:** 1 Department of Health Promotion and Behavioral Sciences, UTHealth Houston School of Public Health, Houston, Texas, United States of America; 2 Department of Public Health, Purdue University, West Lafayette, Indiana, United States of America; 3 School of Social Work, University of Minnesota-Twin Cities, Saint Paul, Minnesota, United States of America; University of Connecticut Health Center: UConn Health, UNITED STATES

## Abstract

Social media has become an increasingly important way to seek and share experiences, support, knowledge, and advice during the COVID-19 pandemic. Reddit, a pseudonymous social media platform, was one way that young people interacted during the pandemic. Our study goals were two-fold: (1) to categorize information sought and provided by users of r/saplings, a subreddit devoted to cannabis use and is often used by young people, and (2) to examine if conversations changed during the COVID-19 pandemic. We extracted 213 randomly selected posts and 2,546 related comments across four time periods (before the pandemic, during the first wave, summer, and next fall). We assessed the volume of posts and comments throughout our study period and conducted a qualitative content analysis. Quantitatively, the findings demonstrated an increase in the number of posts and comments throughout the study period. Given the substantial growth in subreddit activity throughout our study period, Reddit may play an increasingly important role in youth socialization related to cannabis. From the content analysis, we identified three major themes: how to acquire cannabis, how to use cannabis, and associated consequences. Reddit-users prioritized certain content in their posts at different stages of the pandemic. ‘Places to acquire’ and ‘future use’ were most common at the beginning of the pandemic, while the theme of ‘consequences’ and the topic of ‘tolerance’ became more prominent during the summer months. The comments to these posts were generally thorough and responsive to the post. Nearly all the information came from opinions or personal experiences. Firstly, our findings suggest that young people viewed Reddit as a viable outlet for conversations about cannabis. Secondly, due to the nature of the peer comments and lack of verifiable information being exchanged, misinformation may still circulate and inadvertently worsen the efforts to reduce cannabis-related harm. Interventions that provide understandable and accurate cannabis-related information in accessible formats may increase young people’s ability to access and practice harm reduction.

## Introduction

For many people, the various COVID-19 safety measures and subsequent disruptions to daily life led to increased psychological distress (e.g., anxiety, depression) [1–9] and maladaptive coping behaviors (e.g., substance use) [10–12]. Some of the disruptions experienced by children and youth included social isolation, school closures, and loss of routine and healthy outlets [13–16]. In June 2020, approximately 25% of individuals aged 18–24 in the United States reported initiating or increasing substance use to cope with pandemic-related stress or emotions [10], which is consistent with prior research following man-made and natural emergencies in various populations [17–22].

Due to limited in-person social opportunities, many adolescents turned to social media to cope with feelings of loneliness and reconnect with like-minded individuals [23–25]. Social media offers a means to interact with others and to seek and share experiences, support, knowledge, and advice [26, 27]. Specifically, pseudonymous media platforms allow users to control self-presentation and self-disclosure, reducing the likelihood of offline harm [27, 28]. These features likely enhance users’ comfort when discussing sensitive topics, such as drug use and mental health [25–27]. Many users share their experiences via social media to seek support, while others ask for advice or share experiences to establish credibility [29]. Thus, these spaces provide a window into people’s experiences and perceptions of these topics [30, 31].

Despite its popularity and users’ reliance on it for information and support, social media has been found to be a primary source of misinformation and unsubstantiated claims on health topics [32]. Recent policy changes in several countries, including the United States, have loosened restrictions on cannabis, making it easier to access and use. While cannabis-users report using it for fun and experimentation, individuals who are new to cannabis may want to use it for various other reasons, such as alleviating anxiety, depression, insomnia, or chronic pain [33]. These individuals may have a plethora of questions regarding the substance, ways to use it, dosing, and more. Because some negativity surrounding cannabis use persists regardless of policy changes [34], individuals may use pseudonymous social media platforms as a primary source to address their questions [35].

Even though cannabis use on its own is not directly associated with death [36], its use is associated with adverse health effects such as increased risk of cardiovascular disease mortality, reduced cognitive function, and increased neuropsychiatric effects, especially among those who are underage [37, 38]. Furthermore, cannabis may also interact with other medications. As with any substance, it generates concerns such as driving under the influence and dependency. Cannabis use disorder is a particular concern among youth with mood disorders as it has been associated with heightened risk of nonfatal self-harm, homicide, death by unintentional overdose, and all-cause mortality [39]. The misinformation posted to social media may also perpetuate positive health claims about cannabis and the denial of associated dangers to cannabis use, which can further harm young people.

Reddit (https://www.reddit.com/) is one social media platform used by young people. On Reddit, users create pseudonymous usernames and interact with others by creating or commenting on posts within topic-driven communities called “subreddits.” Researchers have utilized Reddit to understand users’ experiences with mental health and substance use [29, 40–47]. Recently, several studies have demonstrated that subreddits without age restrictions (i.e., forums not specifically encouraging or forbidding minors) are a rich source of information and discussion on sobriety, harm reduction, and other substance use-related topics. For example, one recent study demonstrated that people experiencing substance use-related stigma seek support on Reddit [48]. Fentanyl, a synthetic opioid that increases the risk of overdose and mortality, is also commonly studied on Reddit. One study found that Reddit was used to discuss harm reduction strategies and anxieties related to fentanyl-contaminated oxycodone [48, 49], while a second focused on a fentanyl-specific subreddit that found that quality of life impairment, polysubstance use, and tolerance/dependence/withdrawal were the most commonly discussed topics [50]. One study specifically focused on how Reddit could provide insight into the lived experiences of substance use during the pandemic [51]. They found across four substance use-related subreddits that pandemic-specific stressors, limited formal support, disruptions to coping strategies, and access to illicit and prescription drugs were commonly discussed [51].

Adolescents have been shown to post on mental health-related subreddits more often during the COVID-19 pandemic events compared to prior to these events [25]. However, few studies have focused on adolescents’ Reddit use for substance use information and help-seeking, and those that have were primarily focused on e-cigarette use [52–54]. Questions remain about what information young people seek pertaining to cannabis via Reddit and whether the type of information sought changed due to the COVID-19 pandemic.

Thus, we sought to understand if and how people use r/saplings, an adolescent-focused subreddit, to discuss cannabis. The study goals were two-fold: (1) to categorize information sought and provided by users of r/saplings and (2) to examine if r/sapling conversations changed during the COVID-19 pandemic. Because many in-person interactions were restricted throughout the COVID-19 pandemic, social media may play a more prominent role in young people’s cannabis-related socialization. Thus, this study may provide important insights into the type of information sought and provided by young people about cannabis. Further, we identify if these interactions changed throughout the pandemic, which may provide additional insights into how young people coped with pandemic-related stressors.

## Material and methods

### Data collection and sample

In December 2020, we extracted all posts (N = 6,584) and comments (N = 63,269) from r/saplings from October 1, 2019, to October 31, 2020. We obtained our data using Pushshift API, a software interface that archives all posts and comments at the time of submission to Reddit [55]. We chose r/saplings due to its connection with the subreddit r/trees. r/trees is a large, active subreddit surrounding cannabis discussion. This subreddit enforces a set of rules to protect their community and includes a rule that prohibits posting by minors (18 years and younger) (https://www.reddit.com/r/trees/) [56]. From r/trees’ Rules, minors are directed to post on r/saplings instead, a subreddit described as “a place to learn about cannabis use and culture” (https://www.reddit.com/r/saplings/). It is estimated that r/saplings had 53,000 members in October 2019 and 62,000 members in October 2020, as listed on the r/saplings page.

To identify a feasible sample for content analysis, we randomly selected one post per day during four different times: 1) Pre-pandemic (October 1, 2019, to October 31, 2019, and February 1, 2020, to February 29, 2020) to capture posts prior to the pandemic; 2) First Wave (March 1, 2020, to April 30, 2020) to capture posts when lockdown was in place for most countries; 3) Summer (June 1, 2020, to July 31, 2020) to capture posts as lockdown orders were being lifted; and 4) Next School Year (October 1, 2020, to October 31, 2020) to capture posts when young people were returning to school with some COVID-19 safety measures implemented at many institutions. This timeframe was determined according to a couple of deciding factors. In 2020, most Reddit website traffic came from the United States (U.S.) [57]. Therefore, COVID-19 events as they unfolded in the U.S. and were reported by the World Health Organization (WHO) were used to pinpoint which months related to which phases of the pandemic [58]. Since not all major pandemic-related events happened within the same day for some countries, additional months were selected to capture possible variation.

Furthermore, we examined the conventional school schedules of the top ten countries that visited Reddit.com in 2020 to identify which months most often correspond to active school sessions. Since data was collected in December 2020, only October 2020 was selected for ‘Next School Year’, allowing for potential comparison between ‘Pre-pandemic’ and ‘Next School Year.’ This approach yielded a total of 213 posts and all related comments. Posts and comments removed by Reddit moderators or posters before data collection were not replaced.

### Data analysis

First, we plotted the overall number of posts and comments made each month to identify how the number of posts and comments changed during our study period. Then, using the randomly selected dataset described in the Sample section, we conducted a qualitative content analysis using an adaptation of grounded theory to develop the codebook [59]. First, three coders (all of whom are co-authors) independently reviewed all content from the 213 posts and related comments (*n* = 2,546) from r/saplings. Then, we discussed potential patterns during a second review, concentrating on recurring themes and areas of divergence across conversations to draft the initial codebook. After finalizing this draft, we applied the codes to 10% of the dataset and compared the results. We refined the codebook to increase intercoder reliability and ensure the representation of relevant materials. We repeated this process until we reached 90% agreement across all coders and then coded the entire dataset.

To analyze the coded materials, we conducted bivariate analyses to examine changes in the quantity and content of posts and comments across the four time periods. Although we considered applying statistical inference tests, the nuance within our coding structure created very small cell sizes, substantially reducing the tests’ power and interpretability. We also reviewed the materials within each code to identify representative quotes. We paraphrased these quotes, which is in line with ethical guidance and recent conventions when using Reddit data which are in place to reduce the risk of reverse identification [60].

### Ethical issues

We only used publicly available information in this study and complied with the terms and conditions during data collection. Reddit is a pseudonymous platform, meaning individuals may be identified across time through usernames, but their true identity is unknown. The Purdue University Institutional Review Board determined this work was exempt from informed consent, which is consistent with generally accepted ethical guidelines [61, 62].

## Results

**Fig 1:**
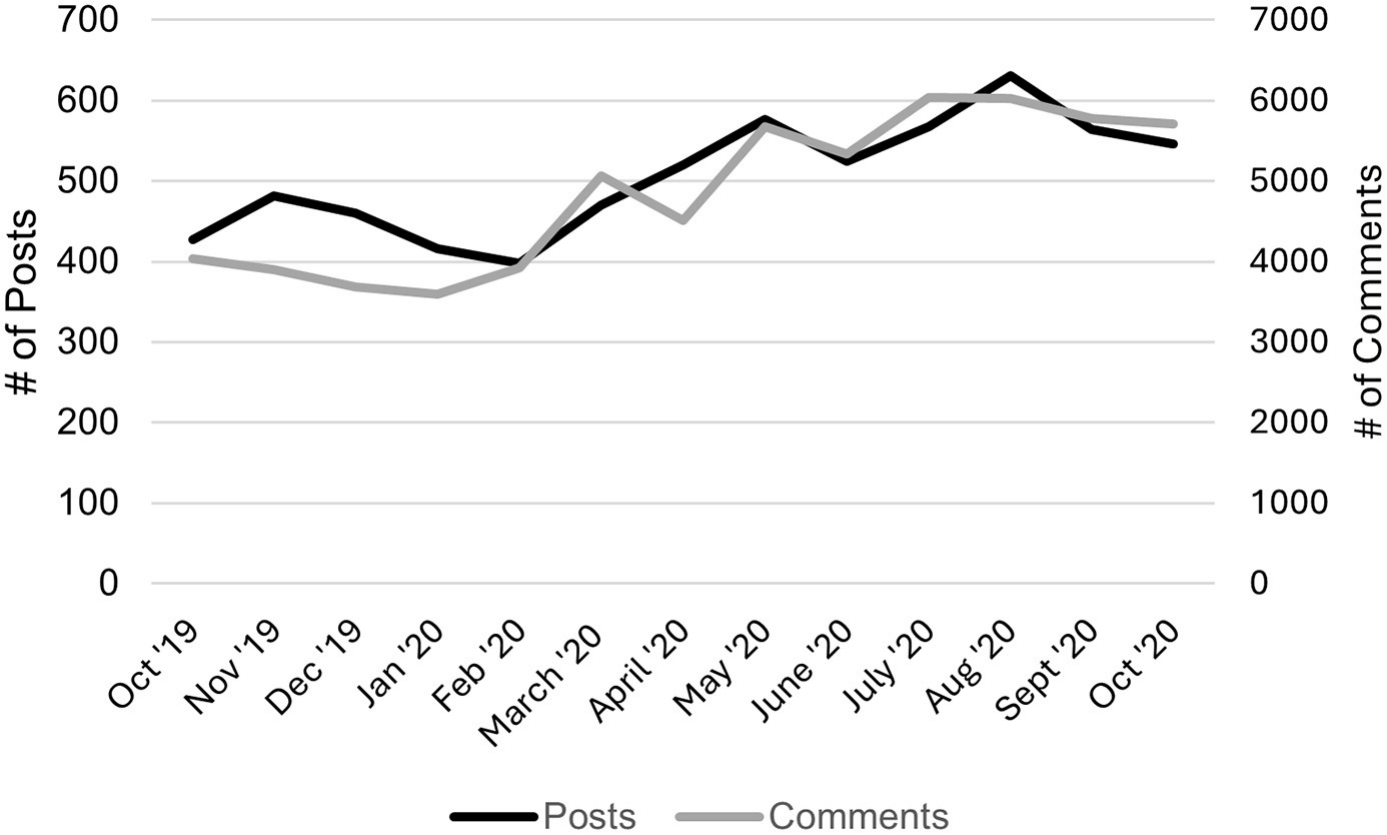
Number of posts and comments on r/saplings during the study period.

The number of posts and comments on r/saplings increased substantially throughout the study period (Fig 1). Posters generally sought advice about cannabis, although some shared their experiences or asked about the experiences of others (Table 1). Eighty-five percent of comments were made by users other than the original poster or the user who created the initial post. Most comments contained detailed, relevant information or follow-up questions.

**Table 1:** Characteristics of posts (n = 213) and comments (*n* = 2,546) in the sample.

	*n* (%)
**Purpose of the Post**	
Seeking Advice	146 (68.5)
Sharing Own Experiences	54 (25.4)
Seeking Others’ Experiences	3 (14.1)
Other	11 (5.2)
**Comment Respondent Role**	
Original Poster	391 (15.9)
Other Respondent	2,063 (84.0)
**Type of Comment** ^a^	
Detailed/Engaged	2,190 (86.0)
Irrelevant	380 (14.9)
Follow-Up to Prior Comment	303 (11.8)
Brief	53 (2.1)
Deleted	53 (2.1)

^a^ Categories are not mutually exclusive, so percentages sum to greater than 100.

We identified three broadly defined themes among posts: (1) how to acquire cannabis, (2) using cannabis, and (3) related consequences (Table 2). Within the theme of ‘acquiring’, users posted content focused on people (e.g., dealers, friends, family), places (e.g., physical locations) (n = 31), and means (e.g., buying, receiving as a gift, stealing) for obtaining cannabis (n = 21), and few posters asked about growing cannabis. The majority of posts involved using cannabis (i.e., the ‘using’ theme) (n = 212), and many posters shared or asked for advice about past (n = 64) or future experiences (n = 83). They often wanted to know if their experiences while high were normal. Posters who recently began using or changed their method of use (e.g., changing from edibles to vaping) inquired about dosage and how to increase the intensity of their high. Others had questions about equipment, the appearance of cannabis, or tolerance. Additionally, some posters shared or asked advice on consequences (i.e., the ‘consequences’ theme), such as cannabis’s effects on health and safety, employment, eviction, or getting in trouble with parents at 48 posts out of 213. Health questions were often related to early age initiation or combining cannabis with prescription medication (e.g., anxiety medications). Sometimes, users specifically discussed how the pandemic was influencing their cannabis use (‘*my parents are staying home due to COVID-19 which sucks because I can’t smoke now*)’. Pandemic-focused posts were most common in the conversations about acquiring cannabis (i.e., previously bought at school or public places), although they also came out in conversations about cannabis use.

**Table 2:** Frequency and content of posts to r/saplings before and during the COVID-19 pandemic.

	Total	Before Pandemic (n = 60)	First wave (n = 61)	Summer (n = 61)	Next Fall (n = 31)	Example Quote
	*n*	*n* (col %)	*n* (col %)	*n* (col %)	*n* (col%)	
**Acquiring Cannabis**						
Places to Acquire	31	4 (6.7)	12 (19.7)	6 (9.8)	9 (29.0)	*Where can I buy prefilled disposable pens online*?
Ways to Acquire	21	3 (5.0)	8 (13.1)	6 (9.8)	4 (12.9)	*I got some edibles from my aunt…*
Growing Cannabis	8	5 (8.3)	1 (1.6)	2 (3.2)	0 (0.0)	*Is my plant a hermaphrodite*?
**Using Cannabis**						
Future Use	83	21 (35.0)	26 (42.6)	20 (32.8)	16 (51.6)	*I have a bunch of different strains on me*.*… Which one should I choose to use while gaming games with my friends*?
Past Experiences	64	18 (30.0)	13 (21.3)	23 (37.7)	10 (32.3)	*Last night I smoked a specific strain of marijuana and had a bad experience*.
						*I couldn’t walk*, *and my hands were shaking…*
Equipment	32	6 (10.0)	11 (18.3)	7 (11.5)	8 (25.8)	*I looked at herb vaporizers but how do I load them*? *Does anyone have any vape recommendations*?
Cannabis Appearance	20	10 (16.7)	4 (6.6)	4 (6.6)	2 (6.5)	*How does this look*? *It’s been in the jar for a few weeks*.
Tolerance	13	4 (6.7)	2 (3.3)	6 (9.8)	1 (3.2)	*Reverse Tolerance*: *Any reason why I’m getting way higher than normal with the usual weed/amount*?
**Consequences**	48	11 (18.3)	11 (18.3)	20 (32.8)	6 (19.4)	*Is using a plastic water bottle bong unsafe*?

Note: Categories are not mutually exclusive, so percentages do not sum to 100.

Users were responsive to posts, with an average of 12.0 (*sd* = 20.2) comments per post. One post received over 200 comments, and only 25 (11.7%) posts received no comments. Generally, comments were thorough and responsive to the materials shared in the post. Most often, the comments were based on opinions or personal experiences. Although rare, some referenced or directed individuals to other sources (e.g., YouTube, websites); of these, only two comments referenced a credible source (e.g., public health department).

The types of posts and comments remained relatively stable over time (Tables 2 and 3). However, there were some meaningful changes. Almost 10% of pre-pandemic posts referenced growing cannabis, compared with less than 3% during other periods. Conversely, posts about possible sources and ways to acquire cannabis became far more common during the pandemic. This increase was especially noticeable between the pre-pandemic and first wave time points (e.g., 4 versus 12 posts for places to acquire, and 3 versus 8 posts for ways to acquire). Discussing places to acquire and future use was common across all periods but was particularly prominent during the first wave of the pandemic and the fall of 2020. Tolerance-related conversations were highest during the summer with six posts, and lowest during the first wave and the fall with 4 and 1 post. Consequences also became the focus among posts in the summer months.

**Table 3:** Average frequency per post and content of comments to r/saplings before and during the COVID-19 pandemic.

	Overall	Before Pandemic	First wave	Summer	Next Fall	Example Quote
	mean (SD)	mean (SD)	mean (SD)	mean (SD)	mean (SD)	
Personal Experiences	2.5 (4.8)	2.0 (3.3)	2.2 (4.6)	3.4 (6.0)	2.2 (5.0)	*As someone who kept both my boyfriend’s and my stash and ended up consuming two-thirds of it on my own*.. *separate that stuffs*
Opinion	7.3 (13.9)	8.4 (20.1)	6.7 (11.2)	6.0 (7.7)	8.3 (14.1)	*I agree with some of the stuff being said about age and waiting*, *I can’t tell you what to do with your life*.
Credible Sources	0.0 (0.1)	0	0.1 (0.2)	0	0	*According to the state’s dep*. *Of health*, *“Cancer*, *human immunodeficiency virus (HIV)*, *multiple sclerosis*, *epilepsy or other seizure disorder*, *or spasticity disorders*.*” are qualifying conditions*.
Other Sources	0.2 (0.6)	0.2 (0.4)	0.3 (0.9)	0.2 (0.4)	0.2 (0.5)	*Some people I know said that they failed the first test and had to go for a second one*.
Directing to Other Resources	0.2 (0.6)	0.2 (0.5)	0.5 (0.9)	0.2 (0.5)	0.3 (0.6)	*I recommend checking out CG Kid on YouTube*.
Brief Support for Earlier Comment	0.2 (0.5)	0.2 (0.5)	0.1 (0.4)	0.1 (0.2)	0.4 (0.8)	*I agree with everything*
Brief Opposition to Earlier Comment	0.1 (0.2)	0.1 (0.3)	0.1 (0.1)	0.1 (0.2)	0	*Absolutely not*. *Next question*.

## Discussion

Our first aim was to categorize information sought and provided by Reddit users on r/saplings and identified three broadly defined themes among posts: (1) how to acquire cannabis, (2) using cannabis, and (3) related consequences (Table 2). Throughout the study period, users discussed various aspects of acquiring, using cannabis, and consequences of using marijuana. Typically, they received detailed, on-topic responses to their questions, indicating a level of social support. Some of our findings in r/Saplings are similar to the work of Costello^29^ on r/trees, a subreddit meant for older individuals and cannabis. Users tended to disclose various information about their Cannabis use (e.g., dosage, ways to use cannabis), and while they discussed ‘tolerance,’ we also did not encounter ‘addiction’ as a topic. Lastly, most comments included advice and instructions similar to r/trees. A proportion of comments were in the form of follow-up questions in order to tailor responses, though most of the advice and instruction came from personal experiences and opinions. While some banter was present, which was coded as ‘Other,’ commenters ultimately formed a sense of camaraderie by providing support in the comments. Other prior work has similarly demonstrated that young people use social media as an important source of information and support about sensitive topics [27–29, 47, 63]. Given the increase in subreddit activity and engagement, Reddit may play an increasingly important role in socialization related to cannabis.

Our second aim was to assess the impact of the COVID-19 on Reddit conversation topics and whether certain themes were prioritized. Places to Acquire, Ways to Acquire, Future use, and Equipment related topics rose in volume by the first wave of the pandemic compared to before the pandemic. At this time, only the theme of consequences remained the same, and the remaining topics declined in volume. ‘Places to acquire’ and ‘future use’ were most frequently discussed during the first wave. The first wave includes the timeframe in which stringent COVID-19 mitigation measures such as stay-at-home orders and closures were put in place. In addition, national data found that youth’s substance use declined during the pandemic. Therefore, our findings suggest that adolescents seemingly struggled to locate cannabis and find in-person resources, thus turning to social media platforms such as Reddit. Our work also supports this latter statement quantitively. The number of posts and comments on r/saplings increased during our study period, from before the COVID-19 pandemic until after its onset. This finding is consistent with prior research, which found increased social media use among youth during the pandemic [23–25]. Altogether, our findings suggest that users turned to social media, including Reddit, and viewed Reddit as a viable outlet for conversations and resource about cannabis.

The theme of Consequences and tolerance became much more prominent during the summer months. During this time, most posts focused on maximizing the pleasant experience while minimizing negative consequences. Although some explicitly framed the conversations around safety (e.g., medication interactions, safe dose), others discussed safety more implicitly (e.g., tolerance issues, avoiding panic attacks). Notably, as of early June, COVID-19 restrictions eased due to available testing, news on vaccination circulated, ‘bubble arrangements’ were endorsed, quarantine requirements were less stringent, and some countries opened their borders [64, 65]. Even prior to the pandemic, our findings demonstrated that users wanted information and support on how to use cannabis safely.

Other studies have similarly demonstrated that social media is not only a valuable source of information but also provides support for harm reduction and recovery [66, 67]. Nevertheless, r/saplings may not always be an adequate source of confirmable information on cannabis. Although several posts specifically asked for ‘experts,’ nearly all information provided was based on opinion or personal expertise, which could still have been rooted in misinformation regarding cannabis use. Most users provided support via leaving detailed and engaged comments, however, commenters without medical backgrounds may unintentionally spread misinformation (e.g., inaccurate medical information, provide information from unidentifiable online sources) and potentially leave detrimental opinions (e.g., original poster worried about drug interactions should ‘give it a try’ and ‘see what happens’). Thus, while there is still great value in lived peer experience within the realm of harm reduction [68], this type of information exchange may result in users not always receiving accurate and verifiable harm reduction information and may lead to worse unintended consequences. Interventions that provide understandable and accurate information in accessible formats may increase young people’s ability to access and consequently practice harm reduction strategies. Due to Reddit’s pseudonymous nature and format, it may be one possible tool in such interventions.

There are limitations to consider when interpreting our work. Reddit is pseudonymous, so we do not know who is using r/saplings or their demographic information. Additionally, even though directed to minors, users who are not minors can still post and comment on r/saplings. As such, our findings may not be confined to only represent posts and comments made by people aged 18 or younger. Further, as a qualitative content analysis, all posts and comments could not be incorporated. Although we randomly selected posts to increase the sample’s representativeness, computational analyses of all conversations may identify additional information. Another limitation is ‘chatbots’ and third-party influences, as both can manipulate online conversations and skew perceptions. However, their influence was limited in our data set since obvious bot-content was coded as ‘Other.’ Our data may still be subject to influence from individuals involved third parties promoting cannabis products. Despite these limitations, our work contributes to the small body of work about young people’s discussions about cannabis during COVID-19. Our findings suggest that young people want to reduce harms associated with cannabis use and may be open to harm reduction information if shared on platforms with like-minded individuals in an easily accessible format.

## References

[1] Brooks SK, Webster RK, Smith LE, Woodland L, Wessely S, Greenberg N, et al. The psychological impact of quarantine and how to reduce it: rapid review of the evidence. The Lancet. 2020 Mar 14;395(10227):912–20. doi: 10.1016/S0140-6736(20)30460-8 32112714 PMC7158942

[2] Bu F, Steptoe A, Fancourt D. Who is lonely in lockdown? Cross-cohort analyses of predictors of loneliness before and during the COVID-19 pandemic. Public Health. 2020 Sep;186:31–4. doi: 10.1016/j.puhe.2020.06.036 32768621 PMC7405905

[3] Graupensperger S, Benson AJ, Kilmer JR, Evans MB. Social (Un)distancing: Teammate Interactions, Athletic Identity, and Mental Health of Student-Athletes During the COVID-19 Pandemic. J Adolesc Health. 2020 Nov;67(5):662–70. doi: 10.1016/j.jadohealth.2020.08.001 32943294 PMC7489994

[4] Hamilton JL, Nesi J, Choukas-Bradley S. Teens and social media during the COVID-19 pandemic: Staying socially connected while physically distant [Internet]. PsyArXiv; 2020 [cited 2023 Oct 5]. https://psyarxiv.com/5stx4/

[5] Jia R, Ayling K, Chalder T, Massey A, Broadbent E, Morling JR, et al. Young people, mental health and COVID-19 infection: the canaries we put in the coal mine. Public Health. 2020 Dec;189:158–61. doi: 10.1016/j.puhe.2020.10.018 33249392 PMC7598559

[6] Lee CM, Cadigan JM, Rhew IC. Increases in Loneliness Among Young Adults During the COVID-19 Pandemic and Association With Increases in Mental Health Problems. J Adolesc Health. 2020 Nov;67(5):714–7. doi: 10.1016/j.jadohealth.2020.08.009 33099414 PMC7576375

[7] Luchetti M, Lee JH, Aschwanden D, Sesker A, Strickhouser JE, Terracciano A, et al. The trajectory of loneliness in response to COVID-19. Am Psychol. 2020 Oct;75(7):897–908. doi: 10.1037/amp0000690 32567879 PMC7890217

[8] Marques de Miranda D, da Silva Athanasio B, Sena Oliveira AC, Simoes-e-Silva AC. How is COVID-19 pandemic impacting mental health of children and adolescents? Int J Disaster Risk Reduct. 2020 Dec;51:101845. doi: 10.1016/j.ijdrr.2020.101845 32929399 PMC7481176

[9] Varma P, Junge M, Meaklim H, Jackson ML. Younger people are more vulnerable to stress, anxiety and depression during COVID-19 pandemic: A global cross-sectional survey. Prog Neuropsychopharmacol Biol Psychiatry. 2021 Jul 13;109:110236. doi: 10.1016/j.pnpbp.2020.110236 33373680 PMC7834119

[10] Czeisler MÉ, Lane RI, Petrosky E, Wiley JF, Christensen A, Njai R, et al. Mental Health, Substance Use, and Suicidal Ideation During the COVID-19 Pandemic—United States, June 24–30, 2020. MMWR Morb Mortal Wkly Rep. 2020 Aug 14;69(32):1049–57. doi: 10.15585/mmwr.mm6932a1 32790653 PMC7440121

[11] Graupensperger S, Fleming CB, Jaffe AE, Rhew IC, Patrick ME, Lee CM. Changes in Young Adults’ Alcohol and Marijuana Use, Norms, and Motives From Before to During the COVID-19 Pandemic. J Adolesc Health. 2021 Apr;68(4):658–65. doi: 10.1016/j.jadohealth.2021.01.008 33781471 PMC8345007

[12] Rodriguez LM, Litt DM, Stewart SH. Drinking to cope with the pandemic: The unique associations of COVID-19-related perceived threat and psychological distress to drinking behaviors in American men and women. Addict Behav. 2020 Nov;110:106532. doi: 10.1016/j.addbeh.2020.106532 32652385 PMC7320671

[13] Cardenas MC, Bustos SS, Chakraborty R. A “parallel pandemic”: The psychosocial burden of COVID-19 in children and adolescents. Acta Paediatr. 2020 Nov 1;109(11):2187–8. doi: 10.1111/apa.15536 32799388 PMC7461397

[14] Lindsay S, Ahmed H, Apostolopoulos D. Facilitators for coping with the COVID-19 pandemic: Online qualitative interviews comparing youth with and without disabilities. Disabil Health J. 2021 Oct;14(4):101113. doi: 10.1016/j.dhjo.2021.101113 34083178 PMC8436050

[15] Orben A, Tomova L, Blakemore SJ. The effects of social deprivation on adolescent development and mental health. Lancet Child Adolesc Health. 2020 Aug;4(8):634–40. doi: 10.1016/S2352-4642(20)30186-3 32540024 PMC7292584

[16] Power E, Hughes S, Cotter D, Cannon M. Youth mental health in the time of COVID-19. Ir J Psychol Med. 2020 Dec;37(4):301–5. doi: 10.1017/ipm.2020.84 32611470 PMC7520633

[17] Brown MRG, Agyapong V, Greenshaw AJ, Cribben I, Brett-MacLean P, Drolet J, et al. Significant PTSD and Other Mental Health Effects Present 18 Months After the Fort Mcmurray Wildfire: Findings From 3,070 Grades 7–12 Students. Frontiers in Psychiatry [Internet]. 2019 [cited 2023 Oct 5];10. https://www.frontiersin.org/articles/10.3389/fpsyt.2019.00623 31543839 10.3389/fpsyt.2019.00623 PMC6728415

[18] Chemtob CM, Nomura Y, Josephson L, Adams RE, Sederer L. Substance use and functional impairment among adolescents directly exposed to the 2001 World Trade Center attacks. Disasters. 2009 Jul;33(3):337–52. doi: 10.1111/j.1467-7717.2008.01077.x 19178553

[19] Rohrbach L, Grana R, Vernberg E, Sussman S, Sun P. Impact of Hurricane Rita on Adolescent Substance Use. Psychiatry. 2009 Sep 1;72:222–37. doi: 10.1521/psyc.2009.72.3.222 19821646 PMC2761882

[20] Cepeda A, Valdez A, Kaplan C, Hill LE. Patterns of substance use among Hurricane Katrina evacuees in Houston, Texas. Disasters. 2010 Apr;34(2):426–46. doi: 10.1111/j.1467-7717.2009.01136.x 19863564 PMC3008163

[21] Flory K, Hankin BL, Kloos B, Cheely C, Turecki G. Alcohol and cigarette use and misuse among Hurricane Katrina survivors: psychosocial risk and protective factors. Subst Use Misuse. 2009;44(12):1711–24. doi: 10.3109/10826080902962128 19895302 PMC2782914

[22] Pollice R, Bianchini V, Roncone R, Casacchia M. Marked increase in substance use among young people after L’Aquila earthquake. Eur Child Adolesc Psychiatry. 2011 Aug;20(8):429–30. doi: 10.1007/s00787-011-0192-2 21674248

[23] Lisitsa E, Benjamin KS, Chun SK, Skalisky J, Hammond LE, Mezulis AH. Loneliness among young adults during COVID-19 pandemic: The mediational roles of social media use and social support seeking. Journal of Social and Clinical Psychology. 2020;39:708–26.

[24] Pennington N. Communication outside of the home through social media during COVID-19. Comput Hum Behav Rep. 2021;4:100118. doi: 10.1016/j.chbr.2021.100118 34568638 PMC8452466

[25] Zhang S, Liu M, Li Y, Chung JE. Teens’ Social Media Engagement during the COVID-19 Pandemic: A Time Series Examination of Posting and Emotion on Reddit. Int J Environ Res Public Health. 2021 Sep 25;18(19):10079. doi: 10.3390/ijerph181910079 34639381 PMC8507823

[26] Robitaille C. Networked psychostimulants: a web-based ethnographic study. Drugs and Alcohol Today. 2020 Jan 1;20(1):50–61.

[27] Valkenburg PM, Peter J. Online communication among adolescents: an integrated model of its attraction, opportunities, and risks. J Adolesc Health. 2011 Feb;48(2):121–7. doi: 10.1016/j.jadohealth.2010.08.020 21257109

[28] Andalibi N, Haimson OL, De Choudhury M, Forte A. Understanding Social Media Disclosures of Sexual Abuse Through the Lenses of Support Seeking and Anonymity. In: Proceedings of the 2016 CHI Conference on Human Factors in Computing Systems [Internet]. San Jose California USA: ACM; 2016 [cited 2023 Oct 5]. p. 3906–18. https://dl.acm.org/doi/10.1145/2858036.2858096

[29] Costello KL, Martin JD, Brinegar AE. Online disclosure of illicit information: Information behaviors in two drug forums. Journal of the Association for Information Science and Technology. 2017 Oct;68(10):2439–48.

[30] Rees S, Mian S, Grabowski N. Using social media in safety signal management: is it reliable? Ther Adv Drug Saf. 2018 Aug 9;9(10):591–9. doi: 10.1177/2042098618789596 30283627 PMC6166319

[31] Lamy FR, Daniulaityte R, Sheth A, Nahhas RW, Martins SS, Boyer EW, et al. “Those edibles hit hard”: Exploration of Twitter data on cannabis edibles in the U.S. Drug Alcohol Depend. 2016 Jul 1;164:64–70. doi: 10.1016/j.drugalcdep.2016.04.029 27185160 PMC4893972

[32] Suarez-Lledo V, Alvarez-Galvez J. Prevalence of Health Misinformation on Social Media: Systematic Review. J Med Internet Res. 2021 Jan 20;23(1):e17187. doi: 10.2196/17187 33470931 PMC7857950

[33] Park JY, Wu LT. Prevalence, reasons, perceived effects, and correlates of medical marijuana use: A review. Drug Alcohol Depend. 2017 Aug 1;177:1–13. doi: 10.1016/j.drugalcdep.2017.03.009 28549263 PMC5542049

[34] Reid M. A qualitative review of cannabis stigmas at the twilight of prohibition. Journal of Cannabis Research. 2020 Dec 7;2(1):46. doi: 10.1186/s42238-020-00056-8 33526147 PMC7819345

[35] Hathaway AD. cannabis users’ informal rules for managing stigma and risk. Deviant Behavior. 2004 Nov 1;25(6):559–77.

[36] Rock KL, Englund A, Morley S, Rice K, Copeland CS. Can cannabis kill? Characteristics of deaths following cannabis use in England (1998–2020). J Psychopharmacol. 2022 Dec;36(12):1362–70. doi: 10.1177/02698811221115760 35946604 PMC9716494

[37] Sun Y, Liu B, Wallace RB, Bao W. Association of Cannabis Use With All-Cause and Cause-Specific Mortality Among Younger- and Middle-Aged U.S. Adults. Am J Prev Med. 2020 Dec;59(6):873–9. doi: 10.1016/j.amepre.2020.07.010 33220757

[38] Fugh-Berman A, Wood S, Kogan M, Abrams D, Mathre ML, Robie A, et al. Medical cannabis: Adverse effects of drug interactions [PowerPoint slides] [Internet]. Medical Marijuana Program. Government of the District of Columbia. Department of Health.; 2015. https://doh.dc.gov/sites/default/files/dc/sites/doh/publication/attachments/Medical%20Cannabis%20Adverse%20Effects%20and%20Drug%20Interactions_0.pdf

[39] Fontanella CA, Steelesmith DL, Brock G, Bridge JA, Campo JV, Fristad MA. Association of Cannabis Use With Self-harm and Mortality Risk Among Youths With Mood Disorders. JAMA Pediatr. 2021 Apr 1;175(4):377–84. doi: 10.1001/jamapediatrics.2020.5494 33464286 PMC7816117

[40] Choudhury MD, De S. Mental Health Discourse on reddit: Self-Disclosure, Social Support, and Anonymity. Proceedings of the International AAAI Conference on Web and Social Media. 2014 May 16;8(1):71–80.

[41] De Choudhury M, Kiciman E, Dredze M, Coppersmith G, Kumar M. Discovering Shifts to Suicidal Ideation from Mental Health Content in Social Media. Proc SIGCHI Conf Hum Factor Comput Syst. 2016 May;2016:2098–110. doi: 10.1145/2858036.2858207 29082385 PMC5659860

[42] Garg M, Maralakunte M, Garg S, Dhooria S, Sehgal I, Bhalla AS, et al. The Conundrum of “Long-COVID-19”: A Narrative Review. Int J Gen Med. 2021;14:2491–506. doi: 10.2147/IJGM.S316708 34163217 PMC8214209

[43] Lea T, Amada N, Jungaberle H. Psychedelic Microdosing: A Subreddit Analysis. J Psychoactive Drugs. 2020;52(2):101–12. doi: 10.1080/02791072.2019.1683260 31648596

[44] Meacham MC, Paul MJ, Ramo DE. Understanding emerging forms of cannabis use through an online cannabis community: An analysis of relative post volume and subjective highness ratings. Drug Alcohol Depend. 2018 Jul 1;188:364–9. doi: 10.1016/j.drugalcdep.2018.03.041 29883950 PMC6692176

[45] Meacham MC, Roh S, Chang JS, Ramo DE. Frequently asked questions about dabbing concentrates in online cannabis community discussion forums. Int J Drug Policy. 2019 Dec;74:11–7. doi: 10.1016/j.drugpo.2019.07.036 31400582 PMC6893129

[46] Robitaille C. “This drug turned me into a robot”: an actor-network analysis of a web-based ethnographic study of psychostimulant use. Can J Public Health. 2018 Dec;109(5–6):653–61. doi: 10.17269/s41997-018-0149-z 30465287 PMC6964562

[47] Bunting AM, Frank D, Arshonsky J, Bragg MA, Friedman SR, Krawczyk N. Socially-supportive norms and mutual aid of people who use opioids: An analysis of Reddit during the initial COVID-19 pandemic. Drug Alcohol Depend. 2021 May 1;222:108672. doi: 10.1016/j.drugalcdep.2021.108672 33757708 PMC8057693

[48] Kepner W, Meacham MC, Nobles AL. Types and Sources of Stigma on Opioid Use Treatment and Recovery Communities on Reddit. Subst Use Misuse. 2022;57(10):1511–22. doi: 10.1080/10826084.2022.2091786 35815614 PMC9937434

[49] Arya S, Nagappala S, Krawczyk N, Gi Y, Meacham MC, Bunting AM. Fentanyl in Pressed Oxycodone Pills: A Qualitative Analysis of Online Community Experiences with an Emerging Drug Trend. Subst Use Misuse. 2022;57(13):1940–5. doi: 10.1080/10826084.2022.2120365 36106770 PMC9909751

[50] Kasson E, Filiatreau LM, Kaiser N, Davet K, Taylor J, Garg S, et al. Using Social Media to Examine Themes Surrounding Fentanyl Misuse and Risk Indicators. Subst Use Misuse. 2023;58(7):920–9. doi: 10.1080/10826084.2023.2196574 37021375 PMC10464934

[51] Gage SH, Brewer G, Steen M, Lyons M. Living with Drug Use and Addiction during the COVID-19 Pandemic. Subst Use Misuse. 2022;57(10):1504–10. doi: 10.1080/10826084.2022.2086695 35787226

[52] Brett EI, Stevens EM, Wagener TL, Leavens ELS, Morgan TL, Cotton WD, et al. A content analysis of JUUL discussions on social media: Using Reddit to understand patterns and perceptions of JUUL use. Drug Alcohol Depend. 2019 Jan 1;194:358–62. doi: 10.1016/j.drugalcdep.2018.10.014 30472576

[53] Liu H, Li Q, Zhan Y, Zhang Z, Zeng DD, Leischow SJ. Characterizing Social Media Messages Related to Underage JUUL E-Cigarette Buying and Selling: Cross-Sectional Analysis of Reddit Subreddits. J Med Internet Res. 2020 Jul 20;22(7):e16962. doi: 10.2196/16962 32706661 PMC7400041

[54] Zhan Y, Zhang Z, Okamoto JM, Zeng DD, Leischow SJ. Underage JUUL Use Patterns: Content Analysis of Reddit Messages. J Med Internet Res. 2019 Sep 9;21(9):e13038. doi: 10.2196/13038 31502542 PMC6786857

[55] Baumgartner JM. Pushshift Reddit API Documentation [Internet]. 2023 [cited 2023 Oct 5]. https://github.com/pushshift/api

[56] rules—trees [Internet]. reddit. [cited 2023 Oct 5]. https://www.reddit.com/r/trees/wiki/rules/

[57] Reddit user worldwide 2020, by country [Internet]. Statista. [cited 2023 Oct 8]. https://www.statista.com/forecasts/1174696/reddit-user-by-country

[58] Timeline of WHO’s response to COVID-19 [Internet]. [cited 2023 Oct 9]. https://www.who.int/emergencies/diseases/novel-coronavirus-2019/interactive-timeline

[59] Schreier M. Qualitative Content Analysis in Practice [Internet]. 2012 [cited 2023 Oct 8]. 280 p. https://us.sagepub.com/en-us/nam/book/qualitative-content-analysis-practice

[60] Proferes N, Jones N, Gilbert S, Fiesler C, Zimmer M. Studying Reddit: A Systematic Overview of Disciplines, Approaches, Methods, and Ethics. Social Media + Society. 2021 Apr 1;7(2):20563051211019004.

[61] Eysenbach G, Till JE. Ethical issues in qualitative research on internet communities. BMJ. 2001 Nov 10;323(7321):1103–5. doi: 10.1136/bmj.323.7321.1103 11701577 PMC59687

[62] Salmons J. Doing Qualitative Research Online [Internet]. SAGE Publications Ltd; 2021 [cited 2023 Oct 5]. https://methods.sagepub.com/book/doing-qualitative-research-online

[63] Yoo M, Lee S, Ha T. Semantic network analysis for understanding user experiences of bipolar and depressive disorders on Reddit. Information Processing & Management. 2019 Jul 1;56(4):1565–75.

[64] Han E, Tan MMJ, Turk E, Sridhar D, Leung GM, Shibuya K, et al. Lessons learnt from easing COVID-19 restrictions: an analysis of countries and regions in Asia Pacific and Europe. Lancet. 2020;396(10261):1525–34. doi: 10.1016/S0140-6736(20)32007-9 32979936 PMC7515628

[65] AJMC. A Timeline of COVID-19 Developments in 2020. AJMC [Internet]. 2021 Jan 1 [cited 2023 Oct 9]; https://www.ajmc.com/view/a-timeline-of-covid19-developments-in-2020

[66] Pestana J, Beccaria F, Petrilli E. Psychedelic substance use in the Reddit psychonaut community. A qualitative study on motives and modalities. Drugs and Alcohol Today. 2020 Jan 1;21(2):112–23.

[67] Sowles SJ, Krauss MJ, Gebremedhn L, Cavazos-Rehg PA. “I feel like I’ve hit the bottom and have no idea what to do”: Supportive social networking on Reddit for individuals with a desire to quit cannabis use. Subst Abus. 2017;38(4):477–82. doi: 10.1080/08897077.2017.1354956 28704167 PMC6143175

[68] Van Schipstal I, Mishra S, Berning M, Murray H. Harm Reduction From Below: On Sharing and Caring in Drug Use. Contemp Drug Probl. 2016 Sep;43(3):199–215. doi: 10.1177/0091450916663248 27721525 PMC5046163

